# Avoidance of Novelty Contributes to the Uncanny Valley

**DOI:** 10.3389/fpsyg.2017.01792

**Published:** 2017-10-26

**Authors:** Kyoshiro Sasaki, Keiko Ihaya, Yuki Yamada

**Affiliations:** ^1^Faculty of Science and Engineering, Waseda University, Tokyo, Japan; ^2^Faculty of Arts and Science, Kyushu University, Fukuoka, Japan; ^3^Japan Society for the Promotion of Science, Tokyo, Japan; ^4^Admission Center, Kyushu University, Fukuoka, Japan

**Keywords:** object perception, emotion, behavioral inhibition system, visual cognition, morphing

## Abstract

A hypothesis suggests that objects with a high degree of visual similarity to real humans trigger negative impressions (i.e., the uncanny valley). Previous studies have suggested that difficulty in object categorization elicits negative emotional reactions to enable the avoidance of potential threats. The present study further investigated this categorization-difficulty hypothesis. In an experiment, observers categorized morphed images of photographs and human doll faces as “photograph” or “doll” and evaluated the perceived eeriness of the images. Additionally, we asked the observers to answer questionnaires on behavioral inhibition systems (BIS). The results indicated that individual differences in the BIS score were associated with enhanced eeriness in the objects with a specific human likeness. These findings suggest that the tendency to avoid a potentially threatening novel experience contributes to promoting the perceived eeriness of objects with some degree of visual similarity to real humans.

## Introduction

**FIGURE 1:**
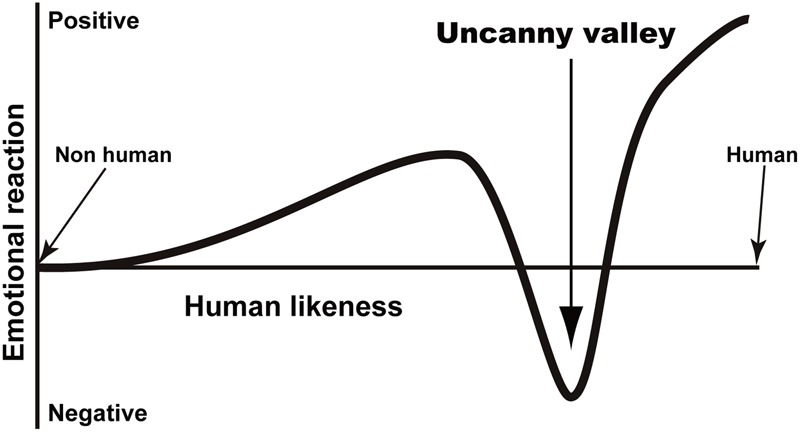
A schematic representation of the uncanny valley.

A primary goal in creating robots is to aid people in real-life situations. Since much of the needed support requires direct interaction between people and robots, people must have a positive attitude toward robots for them to be most effective. One way to achieve this is to design robots to look like people. We often humanize non-human agents (anthropomorphism) and are then motivated to interact with the humanized agents (Epley et al., 2007). For example, when we encounter an object that has eye-like parts, we often share their (illusory) attentional direction (Takahashi and Watanabe, 2013). In addition to appearance, robot movements and voices have also been designed to resemble ours. However, a previous thought experiment proposed a serious obstacle called “the uncanny valley,” which can prevent effective human–robot interaction (**Figure 1**; Mori, 1970, 2012). The uncanny valley refers to the hypothetical changes in subjective robot eeriness with regard to their resemblance to people. At first, as this resemblance increases, the robots appear more familiar. Then, at a certain level of resemblance, the robots appear very eerie. However, when the resemblance becomes sufficiently high, their eeriness declines, and their likeability increases. According to the uncanny valley hypothesis, healthy people are generally situated at the second peak in **Figure 1**, while dead people are located at the bottom of the valley. Moreover, Mori indicated that movement exaggerates the peak and bottom of an emotional response. Although there are both supporting and opposing evidence (Hanson, 2006; MacDorman, 2006; Bartneck et al., 2007; Yamada et al., 2013; Cheetham et al., 2014, 2015; Burleigh and Schoenherr, 2015), developing a way to bridge this valley is important if we want to have a future in which robots are properly integrated into our society.

The uncanny valley has been empirically investigated using several types of stimuli, including pictures of androids (MacDorman, 2005; Saygin et al., 2012; Poliakoff et al., 2013), computer-generated images (Steckenfinger and Ghazanfar, 2009; Lewkowicz and Ghazanfar, 2011; Burleigh et al., 2013), morphed images (Hanson, 2006; MacDorman and Ishiguro, 2006; Seyama and Nagayama, 2007; Cheetham et al., 2011; Yamada et al., 2013), video clips (Ho and MacDorman, 2010), combinations of faces and voices (Mitchell et al., 2011), and actual robots (Yamamoto et al., 2009). The previous studies mainly manipulated the human likeness of these stimuli in a stepwise fashion and measured the perceived eeriness or likeability of the stimuli at each human-like level using a rating method.

While the nature of stimuli that induce the uncanny valley has received much attention, the relationship between the observer’s personality traits and the uncanny valley has not. Indeed, to the best of our knowledge, only one study has examined this issue (MacDorman and Entezari, 2015). Since the uncanny valley results from the direct relationship between agents and observers, experiments that focus merely on stimulus-related factors are not sufficient. Researchers looking into this phenomenon must also investigate observer-related factors, the most important of which are probably individual differences in perceived eeriness. Elucidating individual differences may help generate a deeper understanding of the underlying mechanisms of behavior and cognition (Kanai and Rees, 2011).

The present study focused on the relationship between individual differences in perceived eeriness and trait avoidance of novel experiences. We predicted that these measures would be positively correlated with people who frequently avoid novel experiences, thereby perceiving hard-to-categorize stimuli as eerier than those who do not avoid novel experiences. This is based on our previous findings that, when objects (persons, dogs, and fruits) are hard to categorize, they are perceived negatively owing to a decreased processing fluency (Yamada et al., 2012, 2013, 2014; however, see Looser and Wheatley, 2010; Cheetham et al., 2014, 2015; Burleigh and Schoenherr, 2015; MacDorman and Chattopadhyay, 2016). According to the categorization-difficulty hypothesis, when observers see the uncanny valley stimuli, a hard-to-categorize point of an object (i.e., the point at which it is difficult to discriminate the category of the object as a non-dominant agent) becomes more similar to a dominant agent, and shared visual features increase. Therefore, categorization takes a long time with hard-to-categorize objects (e.g., Yamada et al., 2013). On the other hand, the point of category boundary is the point where the categorical judgment is divided. Although the hard-to-categorize points are considered to be at or near the point of the category boundary in the studies of the uncanny valley phenomenon, categorical discrimination is often less difficult at the category boundary (e.g., Cheetham et al., 2014). Previous findings (Yamada et al., 2012, 2013, 2014) can be explained by the idea that hard-to-categorize objects sometimes pose a threat to observers, and negative impressions that result in avoidance would serve as a defensive mechanism. Importantly, Yamada et al. (2012) showed that individual differences in food neophobia (which leads to trait avoidance of novel foods) predict the degree to which hard-to-categorize objects are perceived negatively. In line with these studies, the categorization-difficulty hypothesis assumed a two-stage process (for more detailed discussions: Kawabe et al., 2017). In the first stage, if the appearance of the hard-to-categorize objects is improbable, then observers cannot categorize such objects into already acquired classes of objects but categorize them into a novel class. In the second stage, the defensive mechanism of avoiding such strange objects functions, and consequently, the negative emotion is evoked. The present study aimed at examining the second stage of the categorization-difficulty hypothesis in the light of the personality trait of avoiding novelty experiences. Considering the defensive mechanism of avoiding strange objects in the second stage of the categorization-difficulty hypothesis, trait avoidance of novel experiences should predict strong negative impressions of hard-to-categorize agents.

The trait of avoidance of novel experiences has been measured using behavioral inhibition system (BIS) scales (Carver and White, 1994). Gray (1970, 1981, 1982) proposed the Reinforced Sensitivity Theory, which is a model involved with approach-avoidance motivational processes. In this theory, three motivational systems are assumed: the behavioral activation system (BAS), fight–flight system, and the BIS. According to Gray’s studies, the BAS is activated by safe signals, such as reward and the absence of punishment. Activation of BAS is involved with the approach of the desired goal and induction of positive emotion. Moreover, the BAS consists of three factors: Drive, Reward Responsiveness, and Fun Seeking. Based on Carver and White (1994), Drive is the factor of persistent pursuit of desired goals, Reward Responsiveness is the factor of positive response to the existence and anticipation of reward, and Fun Seeking is the factor of immediate access to novel and reward stimuli. On the other hand, the fight–flight system is activated by unconditioned aversive stimuli, and thus, this activation induces avoidance reactions and fear. Moreover, the BIS is activated by novel stimuli, conditioned punishments, and absence of rewards. By this activation, anxiety is evoked, causing avoidance of potential threats. Carver and White (1994) created the BIS/BAS scales, and a Japanese version of this scale has also been developed (Takahashi et al., 2007).

In this study, we focus on the BIS because the uncanny valley involves a negative reaction to hard-to-categorize objects. We used morphed cartoon-human faces (Experiment 1) and morphed doll-human faces (Experiment 2) as stimuli the same way as the previous studies (Seyama and Nagayama, 2007; Yamada et al., 2013). Morphing is beneficial for gradually manipulating the human likeness of the stimuli. Carver and White (1994) showed that individuals with high BIS scores were more nervous than those with low BIS scores when they anticipated receiving punishment. There is evidence that high BIS scorers tend to evaluate facial stimuli as more hostile than low BIS scorers (Knyazev et al., 2008). In a study by Sander et al. (2005), BIS scores were positively correlated with neural activities in the ventromedial prefrontal cortex and occipital cuneus evoked by to-be-attended angry voices. Moreover, activations in the amygdala and hippocampus to threatening stimuli are greater in high BIS scorers (Mathews et al., 2004). These studies suggest that high BIS scorers are sensitive and respond negatively to potential threats. If the hypothesis that the uncanny valley partly results from avoiding strangers (Yamada et al., 2012, 2013, 2014; Kawabe et al., 2017) is true, then individuals with high BIS scores should feel a strong sense of eeriness from hard-to-categorize objects. On the other hand, the effect of BIS would not appear with easy-to-categorize objects because the category of these objects is obvious, and thus these objects are not novel and seen as potential threats. Thus, we examined the relationship between scores on the BIS scale and perceived eeriness.

## Experiment 1

### Methods

#### Participants

One thousand people were recruited online through Yahoo! Crowdsourcing, but only 722 people (mean age ± SD: 39.65 ± 9.61; 434 males and 288 females) performed the experiment to the end. The purpose of the study was not revealed to the participants. The experiment was conducted according to the principles laid down in the Helsinki Declaration. The ethics committees of Kyushu University approved the study protocol (approval number: 2013-008).

#### Stimuli and Procedure

**FIGURE 2:**
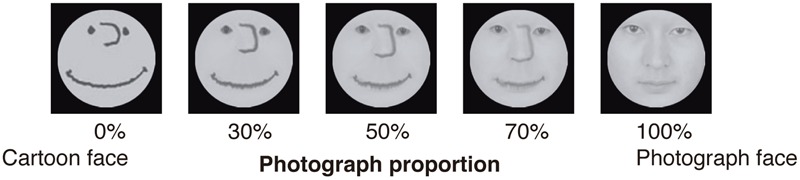
The stimuli used in Experiment 1.

Stimuli consisted of 11 images sized 256 pixels × 256 pixels (**Figure 2**), and the BIS/BAS scale questionnaires were used. The images were same as that of a previous study (Yamada et al., 2013), which was generated by morphing images of a photograph of a cartoon human with that of a human face. A recent study insisted that the stimuli used in Yamada et al. (2013) contained some visual noise (MacDorman and Chattopadhyay, 2016) and this noise induced the negative impression. Concretely, some people might perceive the strong hairline of the 50% image as a scar (please see **Figure 1** of Yamada et al., 2013). To cover this apparent scar, a circular black mask concealed the hair parts and facial contours after morphing. For the questionnaires, we used the Japanese version of the BIS/BAS scales developed by Takahashi et al. (2007). All the items (BIS: 7 items; BAS: 13 items) were scored on 4-point scales ranging from 1 (not at all applicable) to 4 (highly applicable).

The stimuli were presented to the participants on a computer screen. Participants were asked to view the images that were presented one by one and use the 7-point scale to rate the perceived eeriness (1: not at all eerie; 7: very eerie) and categorization confidence (-3: definitely photograph face; 3: definitely cartoon face) of the items without any time limit. The order of the items was randomized across participants. The order of the experimental sessions (image ratings or questionnaires) was also randomized across participants.

#### Data Analysis

We computed Cronbach’s alpha for all the facets of the scales. The sums of BIS, Drive, Reward Responsiveness, and Fun Seeking items were used as the BIS, Drive, Reward Responsiveness, and Fun Seeking scores, respectively. Moreover, we computed the averages of the perceived eeriness and categorical confidences. Additionally, we used the absolute value of the rating scores of the categorical confidence. Higher (lower) values indicated that categorization was easy (difficult). We conducted one-way analyses of variance (ANOVA) on the rating scores of the eeriness and categorical confidence photograph proportion as a within-participant factor. We defined the images whose the rating scores of the categorical confidence were significantly lower than 1.5 as the hard-to-categorize images and the other images as the easy-to-categorize images. Based on this definition, we calculated the average eeriness of the hard-to-categorize images and easy-to-categorize images. We also calculated the average categorical confidence of the hard-to-categorize images and easy-to-categorize images. Our aim was to examine whether the perceived eeriness of the hard-to-categorize objects would be modulated with the levels of the BIS trait. Thus, we performed an analysis of the Spearman rank correlation with the average eeriness of the hard-to-categorize images, the average eeriness of the easy-to-categorize images, the average categorical confidence of the hard-to-categorize images, the average categorical confidence of the easy-to-categorize images, BIS, Drive, Reward Responsiveness, and Fun Seeking scores.

### Results and Discussion

**FIGURE 3:**
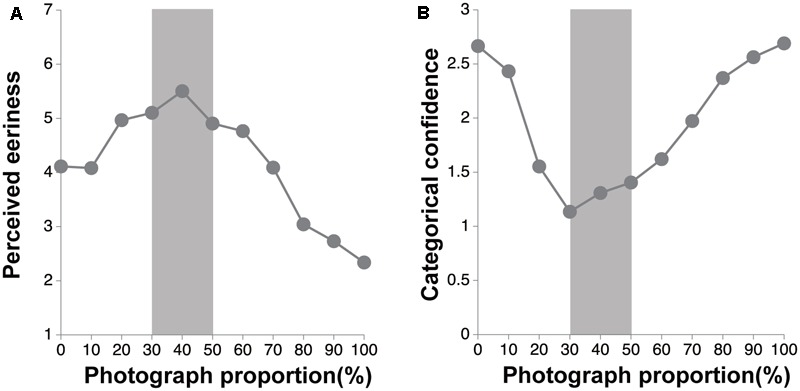
The results of the eeriness evaluation and the categorization tasks. Error bars denote the standard errors of the mean. The gray zone indicates a hard-to-categorize interval (30–50%). **(A)** The perceived eeriness for each photograph proportion. **(B)** The categorical confidence for each photograph proportion.

The Cronbach’s alphas of each facet of the scales were over 0.67. The results of the rating of the perceived eeriness and categorical confidence are shown in **Figures 3A,B**, respectively. We conducted the one-way ANOVA on the rating scores of the perceived eeriness, and categorical confidence showed significant main effects [*F*s(10,7210) > 570.44, *p*s < 0.001, $\eta_{p}^{2}$ s > 0.44]. To confirm whether the rating scores of the categorical confidence at each image were significantly different from 1.5, we conducted *t*-tests. As a results, the rating scores of the categorical confidence were significantly lower than 1.5 at 30, 40, and 50% [30%, *t*(721) = 14.18, *p* < 0.001, Cohen’s *d* = 0.75; 40%, *t*(721) = 1.31, *p* < 0.001, Cohen’s *d* = 0.33; 50%, *t*(721) = 1.31, *p* = 0.002, Cohen’s *d* = 0.16], while they were significantly higher than 1.5 at the other images except 20% [0%, *t*(721) = 50.51, *p* < 0.001, Cohen’s *d* = 2.66; 10%, *t*(721) = 34.09, *p* < 0.001, Cohen’s *d* = 1.80; 60%, *t*(721) = 3.42, *p* < 0.001, Cohen’s *d* = 0.18; 70%, *t*(721) = 13.92, *p* < 0.001, Cohen’s *d* = 0.73; 80%, *t*(721) = 29.25, *p* < 0.001, Cohen’s *d* = 1.54; 90%, *t*(721) = 41.11, *p* < 0.001, Cohen’s *d* = 2.16; 100%, *t*(721) = 50.54, *p* < 0.001, Cohen’s *d* = 2.66]. Thus, we defined the images within 30-50% as the hard-to-categorize images.

The results of the analysis of the Spearman rank correlation are shown in **Table 1**. Importantly, the BIS score was positively correlated with the average eeriness of the hard-to-categorize images (*r* = 0.110, *p* = 0.003) and showed no significant correlation with the average eeriness of the easy-to-categorize images. Moreover, the Reward Responsiveness score was positively correlated with the average eeriness of the hard-to-categorize images (*r* = 0.137, *p* < 0.001) and negatively correlated with the average eeriness of the easy-to-categorize images (*r* = -0.094, *p* = 0.012). On the other hand, the Drive and Fun Seeking scores were not significantly correlated with the average eeriness.

**Table 1:** The results of the perceived eeriness (^∗∗^*p* < 0.01, ^∗^*p* < 0.05).

	EoH	EoE	CCoH	CCoE	BIS	Drive	RR	Fun Seeking
EoH	–							
EoE	0.50^∗∗^(*p* < 0.001)	–						
CCoH	0.09^∗^(*p* = 0.013)	-0.06	–					
CCoE	0.05	-0.22^∗∗^ (*p* < 0.001)	0.54^∗∗^ (*p* < 0.001)	–				
BIS	0.11^∗∗^ (*p* = 0.003)	-0.04	0.13^∗∗^ (*p* < 0.001)	0.18^∗∗^ (*p* < 0.001)	–			
Drive	0.05	-0.02	0.01	-0.01	-0.01	–		
RR	0.14^∗∗^ (*p* < 0.001)	-0.09^∗^ (*p* = 0.012)	0.13^∗∗^ (*p* < 0.001)	0.19^∗∗^ (*p* < 0.001)	0.33^∗∗^ (*p* < 0.001)	0.49^∗∗^ (*p* < 0.001)	–	
Fun Seeking	0.07	0.05	0.03	0.01	0.04	0.46^∗∗^ (*p* < 0.001)	0.47^∗∗^ (*p* < 0.001)	–

*EoH, eeriness of the hard-to-categorize images; EoE, eeriness of the easy-to-categorize images; CCoH, categorical confidence of the hard-to-categorize images; CCoE, categorical confidence of the easy-to-categorize images; RR, reward responsiveness.*

The BIS score was positively correlated with the categorical confidence of the hard-to-categorize images (*r* = 0.131, *p* < 0.001) and easy-to-categorize images (*r* = 0.176, *p* < 0.001). Moreover, the Reward Responsiveness score was positively correlated with the categorical confidence of the hard-to-categorize images (*r* = 0.132, *p* < 0.001) and easy-to-categorize images (*r* = 0.186, *p* < 0.001). On the other hand, the Drive and Fun Seeking scores were not significantly correlated with the categorical confidence of the hard-to-categorize images and easy-to-categorize images.

The perceived eeriness of the hard-to-categorize images increased with the level of the BIS score. These findings suggest that the trait of the avoidance of novelty leads to enhancement in the perceived eeriness of the hard-to-categorize objects. Although we found some relationship between the BAS traits and the perceived eeriness and categorical confidence, we have discussed these issues in the “General Discussion” section.

One might argue that this BIS effect might be stimulus-dependent. Moreover, although the BIS trait was related to the categorical confidence and perceived eeriness of the hard-to-categorize objects, it is unclear whether it was correlated with the categorical boundary. Furthermore, considering previous studies, which showed that the BIS trait was related to negative mood (e.g., Carver and White, 1994; Aoki et al., 2013) and anxiety (e.g., Carver and White, 1994; Mitchell and Nelson-Gray, 2006; Perkins et al., 2010), the anxiety and mood might also influence the perceived eeriness of the hard-to-categorize objects. Therefore, we used other stimuli, conducted a categorization task by the two-alternative forced choice method, and measured the mood and anxiety in addition to the BIS/BAS.

## Experiment 2

### Methods

#### Participants

One thousand and two hundred Japanese people were recruited online through Yahoo! Crowdsourcing, but only 511 people (mean age ± SD: 39.75 ± 9.38; 282 males, 228 females, and 2 unknown) performed the experiment to the end. The purpose of the study was not revealed to the participants.

#### Stimuli and Procedure

**FIGURE 4:**
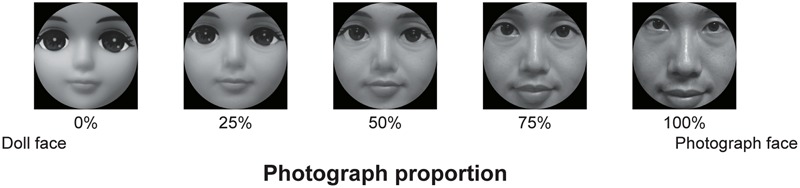
Stimuli used in Experiment 2.

Stimuli consisted of 13 images sized 989 pixels × 1020 pixels (**Figure 4**). The State–Trait Anxiety Inventory (STAI) and Profile of Mood States (POMS) questionnaires were used. The images were generated by morphing the images of a photograph of a human doll face (TOMY Company, Ltd.) with that of a human face. Moreover, to control for the subjective state of the participants, Japanese versions of the STAI (Shimizu and Imae, 1981; Spielberger, 1983) and POMS (Lorr et al., 1971; Yokoyama, 1990) were used. The STAI and POMS items were scored on 4-point scales ranging from 1 (not at all applicable) to 4 (highly applicable) and 5-point scales ranging from 0 (not at all) to 4 (extremely), respectively.

The experiment was conducted online just as Experiment 1. The experiment consisted of three blocks: evaluation, categorization, and questionnaire. The order of the blocks was randomized for all participants. The evaluation block was identical to Experiment 1, except that we asked the participants to evaluate the perceived eeriness of the images and each participant performed 13 trials with 13 morphed images.

In the categorization block, the participants began each trial by pressing a space key. After a blank from 800 to 1200 ms, the stimuli were presented until the participants made a response, after which the participants were asked to categorize the morphed image into one of the two categories (human or doll) by pressing the assigned keys as quickly as possible while maintaining accuracy. If the participants perform categorization for each stimulus only once, the data should be susceptible to the noise. Therefore, in a way similar to that of a previous study (Yamada et al., 2013), each of 13 stimuli was presented 10 times in the categorization task for a total of 130 trials. The trial order was randomized for all the participants. Before the test trial, the participants performed a practice phase in which the procedure was identical to the test trial except that there were five trials with five images (0, 25, 50, 75, and 100%) used in the test trial. The trial order of the practice phase was also randomized for all the participants.

In the questionnaire block, there were three phases: the BIS/BAS, POMS, and STAI. All items on each questionnaire were presented at each phase. The participants answered each item using the mouse, and the order of the phases and items was fixed. Rapid responses were not required.

#### Data Analysis

The data analysis was identical to that of Experiment 1 except the following. We analyzed the reaction time (RT) for categorization and proportion of “photograph” responses on each photograph proportion. After we excluded the outlier data from the trials in which the RT was outside the mean ± 3 SD (Ziegler et al., 2001; Cohen and German, 2010; Constable et al., 2011; Chuang et al., 2015; Huber et al., 2015; Takahashi et al., 2015), we calculated the average RT of the categorization of each image. We performed a one-way ANOVA on the RT and defined the images whose RT was significantly longer than those of 0 and 100% as the hard-to-categorize images. We also calculated the average proportion of “photograph” responses on each image and performed a one-way ANOVA on the proportion of “photograph” responses. Moreover, we calculated a point of subjective equality (PSE) for each participant by fitting a cumulative Gaussian function to the proportion of the photograph responses as a function of the photograph proportion. Additionally, we calculated the average RT of the hard-to-categorize images and easy-to-categorize images. Furthermore, we used Spearman rank correlation to analyze the average eeriness of the hard-to-categorize images and easy-to-categorize images, average RT of the hard-to-categorize images and easy-to-categorize images, BIS, Drive, Reward Responsiveness, Fun Seeking, State STAI, Trait STAI, and POMS^1^ scores.

### Results and Discussion

**FIGURE 5:**
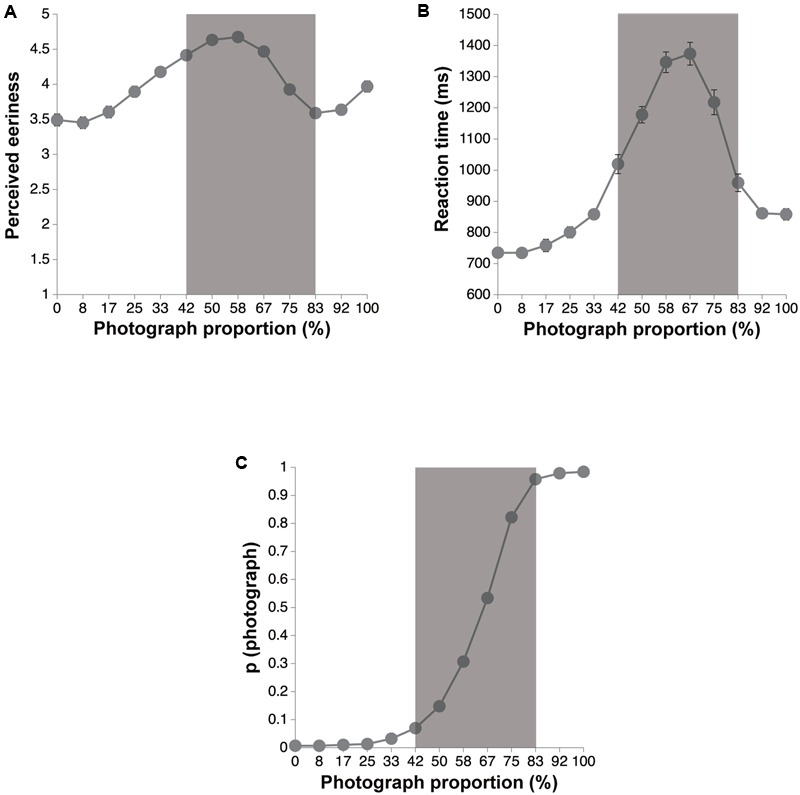
The results of the eeriness evaluation and the categorization tasks. Error bars denote the standard errors of the mean. The gray zone indicates a hard-to-categorize interval (42–83%). **(A)** The perceived eeriness for each photograph proportion. **(B)** Mean RT as a function of photograph proportions. **(C)** The proportions of photograph responses as a function of photograph proportion.

Two participants lost control of the key response, and thus we excluded their data. Moreover, 2% of trials were excluded as outliers. Cronbach’s alpha for all the facets in the scales was over 0.73, indicating a good internal consistency. **Figures 5A,B** show the results of the perceived eeriness and RT, respectively, for each stimulus. We performed a one-way ANOVA on the eeriness score with photograph proportion as a within-participant factor, and found a significant main effect [*F*(12,6096) = 56.25, *p* < 0.001, $\eta_{p}^{2}$ = 0.10]. A one-way within-participant ANOVA also performed on RT with photograph proportion as a factor showed a significant main effect [*F*(12,6096) = 190.67, *p* < 0.001, $\eta_{p}^{2}$ = 0.27]. Multiple comparisons using Ryan’s method (Ryan, 1960) revealed that RT for 42–83% was longer than that of both 0 and 100% [*t*s(6096) > 4.31, *p*s < 0.001, Cohen’s *d*s > 0.19]. Therefore, according to Yamada et al. (2013), we defined 42–83% of the images as hard-to-categorize images. We also calculated the proportion of “photograph” responses on each photograph proportion (**Figure 5C**) and performed a one-way ANOVA on the proportion of the photograph responses with photograph proportion as a within-participant factor. The results showed a significant main effect [*F*(12,6096) = 3088.88, *p* < 0.001, $\eta_{p}^{2}$ = 0.86]. Additionally, we calculated a PSE for each participant by fitting a cumulative Gaussian function to the proportion of photograph responses as a function of the photograph proportion. The PSE was at 63.5% (mean ± 95% confidential interval, 62.5–64.5%; mean *R*^2^ = 0.99).

The results of the analysis of the Spearman rank correlation are shown in **Table 2**. Importantly, the BIS score was positively correlated with the average eeriness of the hard-to-categorize images (*r* = 0.090, *p* = 0.042) and was not significantly correlated with the average eeriness of the easy-to-categorize images. The Fun Seeking score was positively correlated with the average eeriness of the easy-to-categorize images (*r* = 0.100, *p* = 0.02) and was not significantly correlated with the average eeriness of the hard-to-categorize images. Moreover, the Trait-STAI score was positively correlated with the average eeriness of the hard-to-categorize images and was not significantly correlated with the average eeriness of the easy-to-categorize images. Additionally, the POMS score was positively correlated with the average eeriness of the hard-to-categorize images (*r* = 0.124, *p* = 0.005) and the easy-to-categorize images (*r* = 0.138, *p* = 0.002). On the other hand, Drive, Reward Responsiveness, and State-STAI scores were not significantly correlated with the average eeriness.

**Table 2:** The results of the perceived eeriness (^∗∗^*p* < 0.01, ^∗^*p* < 0.05).

	EoH	EoE	RToH	RToE	PSE	BIS	Drive	RR	Fun seeking	State STAI	Trait STAI	POMS
EoH												
EoE	0.63^∗∗^ (*p* < 0.001)	–										
RToH	-0.18^∗∗^ (*p* < 0.001)	-0.20^∗∗^(*p* < 0.001)	–									
RToE	-0.11^∗^ (*p* = 0.01)	-0.15^∗∗^ (*p* = 0.001)	0.81^∗∗^ (*p* < 0.001)	–								
PSE	0.08	0.01	-0.08	0.16^∗∗^ (*p* < 0.001)	–							
BIS	0.09^∗^ (*p* = 0.042)	0.04	0.06	0.05	-0.12^∗∗^ (*p* = 0.006)	–						
Drive	0.04	0.06	-0.05	-0.06	0.05	0.12^∗∗^ (*p* = 0.009)	–					
RR	0.04	0.00	-0.01	-0.02	0.02	0.26^∗∗^ (*p* < 0.001)	0.49^∗∗^ (*p* < 0.001)	–				
Fun seeking	0.06	0.10^∗^ (*p* = 0.02)	-0.14^∗∗^ (*p* = 0.002)	-0.10^∗^ (*p* = 0.03)	0.01	-0.01	0.45^∗∗^ (*p* < 0.001)	0.48^∗∗^ (*p* < 0.001)	–			
State STAI	0.06	0.08	0.00	0.02	-0.01	0.40^∗∗^ (*p* < 0.001)	-0.20^∗∗^ (*p* < 0.001)	-0.14^∗∗^ (*p* = 0.002)	-0.02	–		
Trait STAI	0.09^∗^ (*p* = 0.045)	0.07	0.00	0.03	-0.05	0.67^∗∗^ (*p* < 0.001)	-0.21^∗∗^ (*p* < 0.001)	-0.01	0.01	0.80^∗∗^ (*p* < 0.001)	–	
POMS	0.12^∗∗^ (*p* = 0.005)	0.14^∗∗^ (*p* = 0.002)	-0.08	-0.03	-0.05	0.53^∗∗^ (*p* < 0.001)	-0.14^∗∗^ (*p* = 0.002)	-0.02	0.10^∗^ (*p* = 0.020)	0.78^∗∗^ (*p* < 0.001)	0.84^∗∗^ (*p* < 0.001)	–
												

*EoH, eeriness of the hard-to-categorize images; EoE, eeriness of the easy-to-categorize images; RToH, reaction time of the hard-to-categorize images; RToE, reaction time of the easy-to-categorize images; RR, reward responsiveness.*

The Fun Seeking score was negatively correlated with the average RT of the hard-to-categorize images (*r* = -0.138, *p* = 0.002) and easy-to-categorize images (*r* = -0.096, *p* = 0.03). However, the other scores did not significantly correlate with the average RT. Moreover, the BIS scores were negatively correlated with the PSE (*r* = -0.123, *p* = 0.006), while the other scores did not significantly correlate with the PSE.

Just as Experiment 1, the BIS trait was significantly correlated with the perceived eeriness of the hard-to-categorize objects in Experiment 2. This experiment used the different stimuli from those of Experiment 1. Thus, the results suggest that the perceived eeriness of the hard-to-categorize objects was modulated by the BIS trait independently of the stimuli used in the experiments.

Trait anxiety was also significantly correlated with the perceived eeriness of the hard-to-categorize objects. This is consistent with the previous study (MacDorman and Entezari, 2015) showing that the perceived eeriness of androids relates to trait anxiety. Given that the perceived eeriness of the easy-to-categorize objects were not related to trait anxiety, the trait anxiety possibly increased the perceived eeriness of the human-like objects. Moreover, the POMS also influenced the perceived eeriness and the RT, but this effect was found in the case of most of the images, and not just the hard-to-categorize images. This can be explained by the mood-congruency effect (e.g., Bower, 1981).

The BIS trait also influenced the PSE but not the RT in Experiment 2. The modulation in the PSE indicates that the high level of the BIS trait biased the categorical boundary toward the photograph. Additionally, Fun Seeking scores were related to the perceived eeriness and RT. We have discussed these issues in General discussion in conjunction with the results of Experiment 1.

## General Discussion

As BIS is a signal-driven danger system that controls avoidance of novel experiences, we investigated whether individual differences in BIS scores affected perceived eeriness of the hard-to-categorize objects. In both the present experiments, we used the BIS scale (Carver and White, 1994; Takahashi et al., 2007) and found that the BIS scale scores significantly affected the perceived eeriness when the images were hard-to-categorize. Therefore, the present results suggest that the individual personality trait associated with the avoidance of novel experiences is associated with a steeper uncanny valley.

The present study also showed that the BIS trait was related to the categorization task. In sum, the BIS trait was positively correlated with the categorical confidence in Experiment 1. Moreover, the PSE was shifted to the photograph face as the BIS scores increased, but the BIS scores had no impact on the RT in Experiment 2. Generally, the confidence and speed of a judgment task negatively correlate with each other (e.g., Johnson, 1939; Festinger, 1943; Vickers and Packer, 1982; Baranski and Petrusic, 1998) and like these previous studies, higher confidence should lead to a faster judgment of categorization in the present study. Hence, if the effect of BIS on the categorization confidence as shown in Experiment 1 also occurs on judgment speed, the BIS score would negatively correlate with the RT in Experiment 2. However, this was not the case. The results of Experiment 2 showed no significant correlation between the RT and BIS score. At this time, it is difficult to specify the reason for this dissociation between the categorical confidence and RT because the categorization tasks (rating vs. binary judgment) and stimuli (object, category, the number of morphing steps, etc.) were different in Experiments 1 and 2. However, at the least, the dissociation possibly indicates that the effect of BIS on categorization is not robust. Moreover, we found that the BIS trait modulates the categorical boundary in Experiment 2. This is novel, and there is no clear reason for this shift of the boundary as yet. One of the possibilities is that the high BIS scorers tend to judge ambiguous objects as more dangerous among those in the acquired categories (i.e., doll and human in Experiment 2). This notion is consistent with the Error Management Theory (e.g., Haselton and Buss, 2000). Perhaps, the participants might regard humans as more dangerous than the doll because the doll does not move and thus is less likely to be harmful. As a result, the categorical boundary possibly shifted toward the human. These issues of the effect of the BIS trait on categorization should be more directly and systematically examined in the future studies.

How did the BIS trait influence the perceived eeriness of the hard-to-categorize objects? It is possible that the BIS trait modulates the degree of perceived eeriness of the eerie objects. Based on the categorical difficulty explanation (Yamada et al., 2012, 2013, 2014; Kawabe et al., 2017), if hard-to-categorize objects seem to be improbable, then they cannot be categorized into the existing classes but are categorized into a novel class. People then tend to avoid these strange objects based on the defensive mechanism, and hence a negative emotion (i.e., eeriness) is induced. The BIS trait is related to avoiding novel and potential threats, and thus it is very likely that high BIS scorers excessively avoid the hard-to-categorize objects, and hence strongly experience eeriness in comparison to the low BIS scorers.

The present study found a relationship between the BAS traits and the perceived eeriness and categorical performance. However, as the results were inconsistent between the two experiments, it was difficult to reach a clear conclusion. Briefly, although Reward Responsiveness had some correlation with the perceived eeriness and categorization performance in Experiment 1, these correlations were not significant in Experiment 2. On the other hand, Fun Seeking did not involve the perceived eeriness and categorization performance, while it was somewhat correlated with the perceived eeriness and categorization performance. This inconsistency might indicate that the effects of the BAS traits are stimulus-dependent in contrast to the BIS traits. Originally, as mentioned above, the BAS consists of a mixture of multiple factors and is orthogonal to the BIS, and hence these results do not affect the interpretation of the main results of the present study.

The uncanny valley may have several causes. It is likely that many factors acknowledged in neurophysiological (Cheetham et al., 2011; Saygin et al., 2012), cognitive (Seyama and Nagayama, 2007; Mitchell et al., 2011; Yamada et al., 2013), and social approaches (MacDorman and Ishiguro, 2006; MacDorman et al., 2009b) interact and are finally integrated as a conscious perception of eeriness. From this view, these findings can only explain the integrative process of the uncanny valley from cognitive, perceptual, and affective perspectives, but even so, they provide some important clues to other approaches. One of them is that the effect seems to be related to the motivational system. The BIS regulates the motivation to avoid potential threats, and the BAS regulates the approach motivation based on safe signals (Gray, 1970); trait activations of these systems are quantified by the BIS/BAS scales (Carver and White, 1994; Takahashi et al., 2007). In the present experiments, the BIS score was related to the eeriness of hard-to-categorize objects that are potentially threatening. Thus, the avoidance of novelty is an important part of the integrative process that generates the uncanny valley. Moreover, morphing artifacts, which often occur in studies using morphed images, could affect observers’ responses by drawing attention to the noisy parts of an image (Cheetham and Jancke, 2013). A previous study (MacDorman and Chattopadhyay, 2016) pointed out that these morphed artifacts, rather than categorization difficulty, induced perceived eeriness. However, we used morphed images that had no visual artifacts and thus could reject the possibility that the eeriness of hard-to-categorize objects was induced by visual noise in an image (see also Ferrey et al., 2015).

The present study showed that individuals with high BIS sensitivity evaluate hard-to-categorize objects as eerier compared to those with low BIS sensitivity. These findings support those of MacDorman and Entezari’s (2015) study, in which it was revealed that neuroticism and anxiety traits were correlated with the perceived eeriness of androids. In fact, the BIS scores are correlated with scores on both neuroticism (e.g., Carver, 2004) and anxiety (e.g., Carver and White, 1994). The present study also showed a positive correlation between the perceived eeriness and trait anxiety in Experiment 2. Neuroticism is a component of anxiety (Eysenck, 1965), and individuals with high BIS sensitivity are vulnerable to anxiety in certain situations (Fowles, 1987). Additionally, Fowles (1987) suggested that BIS sensitivity is related to the avoidance of anxiety-inducing situations. Therefore, MacDorman and Entezari’s (2015) findings might reflect the possibility that persons with high neuroticism and anxiety have high BIS sensitivity; evaluating hard-to-categorize objects as highly eerie might reflect an avoidance reaction.

The present study used the Japanese version of the BIS/BAS scales (Takahashi et al., 2007) based on Carver and White (1994). However, the revised Reinforced Sensitivity Theory (Gray and McNaughton, 2000) was not reflected in these scales. In an earlier version of the Reinforced Sensitivity Theory (Gray, 1987), the BIS was considered as a system that responded to novel stimuli, conditioned punishments, and absence of rewards. On the other hand, the BIS is activated by a conflict in the choices of response to the presented stimuli, and this activation induces avoidance of potential threats in the revised Reinforced Sensitivity Theory (Gray and McNaughton, 2000). Therefore, the BIS scales might become slightly different if the scales are recreated based on the revised Reinforced Sensitivity Theory. We should examine whether this difference could influence our findings after the new BIS scales are developed.

Previous studies proposed that categorical difficulty induced the eeriness of the hard-to-categorize objects (Yamada et al., 2013; Ferrey et al., 2015). However, there are several studies that do not support this categorical ambiguity theory (Looser and Wheatley, 2010; Cheetham et al., 2014, 2015; Burleigh and Schoenherr, 2015; MacDorman and Chattopadhyay, 2016). What causes these discrepancies? One plausible factor is the stimulus used for morphing. Supporting studies used two kinds of neutral objects (e.g., a real human and a stuffed human) and morphed them. On the other hand, most opposing studies morphed eerie and neutral stimuli. In other words, one side of their stimuli was already in the “valley” of the uncanny valley. Therefore, the peak eeriness point might not coincide with the most hard-to-categorize point in these studies. However, both supporting and opposing studies agree with the notion that categorization ambiguity is not a unitary factor of the uncanny valley (for more detailed discussions, see Kawabe et al., 2017).

Several studies have claimed that the uncanny valley stems from a perceptual mismatch or realism inconsistency; negative emotion could be evoked by an object that has incongruent features (e.g., Brenton et al., 2005; Seyama and Nagayama, 2007; MacDorman et al., 2009a; Mitchell et al., 2011; Kätsyri et al., 2015; MacDorman and Chattopadhyay, 2016). Perceived eeriness induced by hard-to-categorize objects in the present study might also be explained by this perceptual mismatch hypothesis or realism inconsistency hypothesis. This is because, in the image with hard-to-categorize points, the eyes are much larger than the face of a real human. Such a mismatch or inconsistency between realistic and artificial features might be associated with perceived eeriness. However, these hypotheses and the categorization-difficulty hypothesis are not mutually exclusive (see also Kawabe et al., 2017). In the categorization-difficulty hypothesis, the objects are categorized into a novel class based on the appearance improbability. The evaluation of appearance improbability does not deviate from that of the perceptual mismatch or realism inconsistency. That is, there is no contradiction among these three hypotheses in terms of the judgment of the object appearance. Furthermore, the key findings of the present study was concerned with how the object that induces strong eeriness after its appearance is judged as the improbable one; the perceived eeriness of the improbable objects results from an avoidance reaction to the novelty and strangeness of these objects.

One might argue that the sexes of the photograph and doll faces seem to be male and female, respectively, and morphing intersexual images could mediate the present results. In fact, the sex of both the human and doll was male. Moreover, even if the sex of the doll face was perceived as female, a previous study revealed that morphed images of different genders did not cause a negative evaluation (Yamada et al., 2013). Thus, it is less likely that perceived sex influenced our main findings. Additionally, the perceived eeriness of the photograph face was significantly higher than that of the doll face, and thus the perceived attractiveness might also differ between them. It is unclear whether this difference in attractiveness mediated the difference in the perceived eeriness between the degrees of the BIS. Future studies should address these issues.

It is still unclear what emotional processing contributes to the uncanny valley. Recently, we showed that subliminally presented odors mitigate negative impressions of hard-to-categorize foods (Yamada et al., 2014). This may be because these odors unconsciously add information that help categorization. One theory has proposed that emotional processing integrates information from deliberate, conscious pathways with that from automatic, unconscious pathways (LeDoux, 1996, 2012). There is evidence that danger signals are preferentially processed even outside of visual awareness (Liddell et al., 2005; Yang et al., 2007; Tamietto et al., 2009; Yamada and Kawabe, 2011). There is also evidence that BIS scores are related to neural responses (Cools et al., 2005) and gray matter volume (Barrós-Loscertales et al., 2006) in the amygdala, which is thought to be a locus of emotional processing. Previous studies on the uncanny valley have been performed mainly using conscious stimuli. Further research is needed to clarify the unconscious aspects of the uncanny valley.

Until now, we have only investigated the role of categorical processing in object judgment using an explicit categorization task. However, the uncanny valley is an everyday phenomenon that occurs regardless of any explicit cognitive task. As discussed above, determining the degree to which the uncanny valley process occurs automatically and unconsciously is important. Future investigations using incidental learning tasks that have been used primarily in memory (Jones, 1990; Jones and Martin, 1992) and learning research (Reber, 1989; McGeorge and Burton, 1990) may provide insight into this issue. Incidental learning is knowledge acquisition that occurs without intention. It is likely that incidental categorization also produces the uncanny valley for objects that are hard to categorize. We used morphed images developed primarily from photographs and dolls, but the uncanny valley is a phenomenon that also occurs for human-like robots. To apply the categorization-difficulty hypothesis to everyday situations, future studies need to examine it using real robots (MacDorman and Ishiguro, 2006).

## Author Contributions

All of the authors designed the experiment. KS conducted the experiment and analyzed the data. All authors discussed the data and wrote the paper. YY supervised the project.

## Conflict of Interest Statement

The authors declare that the research was conducted in the absence of any commercial or financial relationships that could be construed as a potential conflict of interest.

## Footnotes

1 The POMS consists of six facets: Tension-Anxiety (T-A), Depression-Dejection (D), Anger-Hostility (A-H), Vigor (V), Fatigue (F), and Confusion (C). Five facets, except V, are related to negative mood, and thus, the mood of the participants is more negative when these scores are high. However, V is associated with a positive mood, and hence the mood of the participants is more positive when this score is high. Since we used POMS for assessing the general mood, we subtracted the V scores from the sums of the scores of the five facets (e.g., Cruess et al., 1999; Zubieta et al., 2005; Troisi et al., 2010) to obtain the POMS score.
